# *Streptomyces enissocaesilis* L-82 has broad-spectrum antibacterial activity and promotes growth for *Carassius auratus*

**DOI:** 10.1007/s00253-024-13031-7

**Published:** 2024-02-19

**Authors:** Wensu Long, Wenjuan Zhao, Liangliang He, Tahir Ali Khan, Ximiao Lai, Yunjun Sun, Weitao Huang, Ganfeng Yi, Liqiu Xia

**Affiliations:** 1 State Key Laboratory of Developmental Biology of Freshwater Fish, Hunan Provincial Key Laboratory of Microbial Molecular Biology, College of Life Science, Hunan Normal University, No. 36 Lushan Street, Changsha, 410081 People’s Republic of China; 2 Key Laboratory of Aquatic Functional Feed and Environmental Regulation of Fujian Province, Fujian Dabeinong Aquatic Sci. & Tech. Co, Ltd, Zhangzhou, 363500 China; 3 Fantastic Victory (Shenzhen) Scientific Innovation Group, Co. Ltd, Shenzhen, China

**Keywords:** *Streptomyces enissocaesilis*, Crucian carp, *Aeromonas hydrophila*, Antibacterial active substances

## Abstract

**Abstract:**

*Aeromonas* is the main pathogen causing bacterial diseases in fish. The disadvantages of chemical drugs to control fish diseases have been highlighted, and it is urgent to find an eco-friendly control method. In this study, an actinomycete strain with antibacterial activity against fish pathogenic bacteria was screened from soil samples. Combined with morphological characteristics, physiological and biochemical characteristics, and *gyrB* gene and whole genome comparison analysis, it was identified as a new strain of *Streptomyces* e*nissocaesilis*, named *Streptomyces enissocaesilis* L-82. The strain has broad-spectrum antibacterial activity against fish pathogens. A substance with a mass-to-charge ratio of 227.20 [M + H] ^+^ was isolated and purified by high-performance liquid chromatography and mass spectrometry. It was presumed to be a derivative of 5-dimethylallylindole-3-acetonitrile. The strain is safe and non-toxic to crucian carp, and can stably colonize crucian carp and inhibit the proliferation of *A. hydrophila*. After feeding the feed containing 1 × 10^8^ CFU/mL strain concentration, the weight growth rate and specific growth rate of crucian carp increased, the activity of ACP and SOD in serum increased, and the survival rate of crucian carp increased after challenge. Genome-wide analysis showed that the strain had strong ability to metabolize and tolerate extreme environments. And has a strong potential for disease resistance. Therefore, the strain is expected to be developed as a feed additive for fish farming.

**Key points:**

• *The new Streptomyces enissocaesilis L-82 has a broad spectrum and stable antibacterial activity and meets the safety standards of feed additives.*

• *Strain L-82 can colonize crucian carp, improve the growth, antioxidant, and immune performance of the host, and improve the survival rate after being infected with A. hydrophila.*

• *Genome-wide analysis suggests that the strain has great disease resistance potential and is expected to be developed as a feed additive for fish culture.*

**Supplementary Information:**

The online version contains supplementary material available at 10.1007/s00253-024-13031-7.

## Introduction

Crucian carp (*Carassius auratus*) is popular with consumers because of its delicious meat, abundant in protein, vitamins, unsaturated fatty acids, and other nutrients. This variety has the characteristics of tolerance to low oxygen environment and easy reproduction. At the same time, it has a fast growth rate and high yield, which can bring great economic benefits to farmers.

With the continuous expansion of the scale of fish farming and the promotion of intensive farming models, the gradual increase in population density has also led to the deterioration of the breeding environment, providing a favorable environment for the survival and proliferation of conditional pathogenic bacteria in fish, which led to the outbreak of bacterial diseases in fish. These bacterial diseases will lead to a decrease in fish body weight, and also increase the mortality of fish, which will cause great economic losses to fish farmers. In recent years, in order to meet the increasing demand, farmers have used antibiotics, growth promoters, and other additives to maximize production. Although these measures can indeed improve the yield of fish culture to a certain extent, the application of these chemical drugs to conventional use will lead to serious bacterial diseases of fish and seriously threaten the sustainable development of the fish culture industry (Nayak 2010). Therefore, it is very important to find a scientific prevention and control measure that is conducive to the sustainable development of the fish breeding industry.

Aeromonas is widespread in aquatic ecosystems and is a major pathogen causing Aeromonas mobilis septicemia (MAS) in freshwater fish. *Aeromonas hydrophila*, *Aeromonas veronii*, *Aeromonas sobria*, *Aeromonas caviae*, *Aeromonas salmonicida*, etc. are some of the most common Aeromonas species that can cause the disease, of which *A. hydrophila* is the most toxic (Shirajum Monir et al. 2020). When water pollution, high stocking density, and improper breeding operations increase the risk of sepsis outbreaks. When water pollution, high stocking density, and improper breeding operations increase the risk of sepsis outbreaks (Azzam-Sayuti et al. 2021). The disease can cause swelling, necrosis, ulceration, and hemorrhagic septicemia in fish tissues, and the incidence rate is fast, and the mortality rate is also very high, causing huge economic losses to fish farmers (Li et al. 2021). Therefore, the prevention and control of such diseases is the focus of prevention and control in fish aquaculture.

When probiotics are used as food additives, they can promote food digestion, promote host growth, and improve production efficiency. It can also regulate the host’s immune response and improve the host’s disease resistance and survival rate after infection with bacteria (Chen et al. 2023). In addition, probiotics provide benefits to the host by directly or indirectly stimulating the intestinal flora in the host (Soliman et al. 2019). In 1986, Kosaza discovered that the use of spores of *Bacillus* toyoi as a feed additive could increase the growth rate of yellowtail fish (*Xenocypris davidi*), the first report on the use of probiotics in aquaculture. At present, the most commonly used probiotics are *Lactobacillus*, *Bacillus*, *Streptomyces*, *Microalgae*, and *Yeast* (Son et al. 2009).

*Streptomyces* can produce a large number of active secondary metabolites, including antibiotics, hydrolases, vitamins, and growth promoters. About 60% of all known drugs are produced by *Streptomyces* (Cuozzo et al. 2023; Porter 1971). Dharmaraj et al. (Li et al. 2020) found that the growth performance of swordtail fish (*Xiphophorus maculatus*) was significantly improved after 50 days of continuous feeding with feed supplemented with *Streptomyces*. *Streptomyces* can secrete extra-hydrolytic enzymes to improve the activity of starch hydrolysis and protein hydrolysis in the digestive tract of the host, so as to make more effective use of feed (Cuozzo et al. 2023). Das et al. (2010) added *Streptomyces* to the feed, which increased the hydrolase activity in the digestive tract of *Penaeus orientalis*, helping it to use the feed more effectively and increase the weight of the shrimp. You et al. (2005) reported a *Streptomyces* strain that can produce active substance siderophores and found that the *Streptomyces* can affect the growth of pathogenic *Vibrio* by competing for iron in the aquatic environment. The ability of *Streptomyces* to resist enzymatic digestion and the ability of its spores to resist desiccation can help them resist harsh environments, allowing them to tolerate low pH values in the stomach and intestines of animals (McBride and Ensign 1987). Due to these characteristics, *Streptomyces* has a higher survival rate in the host than traditional probiotics. Therefore, *Streptomyces* can be used as a microbial feed additive to help fish resist bacterial diseases and has great development potential in fish culture.

In this study, a new strain of *Streptomyces enissocaesilis* L-82 (deposited in China Center for Type Culture Collection; CCTCC No.: M20231088) with broad-spectrum antibacterial effect on fish pathogens was screened. This strain can improve the growth and immune performance of crucian carp and enhance the resistance of crucian carp to *A. hydrophila* (deposited in 73 China Center for Type Culture Collection; CCTCC No.: M2014157). This study laid a theoretical foundation for the development of the strain into a microbial feed additive and its application in fish farming to enhance the disease resistance of fish.

## Materials and methods

### Isolation and identification of strain L-82

A proper amount of soil samples from different regions were stirred and mixed with sterile water. After standing and stratification, the supernatant was diluted gradiently by a tenfold dilution method and spread on GAUZE medium respectively. After incubation at 30 °C, the single colonies similar to the typical colony morphology of actinomycetes were selected for purification. The strain L-82 with an antibacterial effect was screened by using fish pathogenic bacteria as indicator bacteria. The morphology of the strain on the plate was observed by coating, Gram staining, and scanning electron microscopy (SEM, Hitachi Su8010, Japan). The physiological and biochemical characteristics of strain L-82 were identified by various physiological and biochemical media. The genomic DNA of the strain was extracted with a bacterial DNA extraction kit and the gryB gene (gyrB F,5′-GAAGTCATCATGACCGTTCTGCAYGCNGGNGGNAARTTYGA-3′; gyrB R,5′-AGCAGGGTACGGATGTGCGAGCCRTCNACRTCNGCRTCNGTCAT-3′) was amplified. The sequencing results were spliced and compared with NCBI. The homologous sequences were downloaded and the phylogenetic tree was constructed by the neighbor-joining method (no. of bootstrap replication 1000) of MEGA X software. Subsequently, the whole genome sequence of the closest strain was downloaded. RAST was used to analyze the sequence similarity and collinearity with the whole genome sequence of strain L-82.

### Antibacterial determination and stability analysis

The strain L-82 was inoculated into an AM3-1 liquid medium (30 °C, 200 rpm) and the fermentation supernatant was collected by centrifugation. The antibacterial activity of the indicator pathogen was detected by punching method. The indicator pathogen was coated on an LB plate, dried, and punched. The 60 μL fermentation supernatant was added to the well, and the pathogen was cultured at 30 °C for 12 h. The pathogen was coated on the LB medium, and the diameter of the inhibition zone was measured with a vernier caliper (0.05 mm).

Take the fermentation broth with the best fermentation time. The fermentation broth was treated with different temperatures and pH conditions for 1 h and then the original conditions were restored. The fermentation broth was irradiated with ultraviolet light (254 nm) at different times. The fermentation broth was treated with protease K at 55 °C, trypsin and pepsin at 37 °C for 1 h, and then treated at 85 °C for 5 min to terminate the enzyme reaction. The antibacterial activity against *A.hydrophila* was detected after different treatments. The diameter of the inhibition zone was measured with a vernier caliper (0.05 mm).

### Biosafety test

In 96-well plates, grass carp liver L8824 cells and HEK293T cells were added to each well at a density of 105 cells/well and incubated in a cell incubator at 30 ℃ and 37 ℃ for 12 h, respectively. The cell-free supernatant of strain L-82 was collected and filtered through a 0.22 μm syringe filter and added to a 96-well plate. The fermentation supernatant of *A. hydrophila* and AM3-1 medium were used as controls. The morphology of L8824 was analyzed and observed by an inverted optical microscope (Leica Microsystems, Italy) at different time periods, respectively, to determine the in vitro cell safety of strain L-82.

At the same time, the crucian carp was continuously fed with a feed containing 1 × 10^8^ CFU/mL strain L-82 at a feeding rate of 1% for 30 days. At the end of the experiment, the control group, the experimental group, and the control group were randomly selected for anatomy. The four tissues of the liver, kidney, spleen, and intestine were taken for HE staining to compare the damage of tissues and further determine the safety of strain L-82 in crucian carp.

### Feeding conditions of crucian carp

Healthy crucian carp were obtained from a breeding base (Changsha, China) and acclimated to experimental conditions in a tank (60 L) for 2 weeks. Ninety crucian carps were randomly divided into two groups (CK and 1 × 10^8^ cfu/g) with 3 replicates in each group. All fish were fed twice a day at 1% of body weight. The temperature of the feeding environment was 25 ± 2 °C, and the water was replaced every 2 days.

### Growth performance analysis

The initial weight (W_0_) and final weight (W_t_) were recorded. Weight gain rate (WGR), specific growth rate (SGR), and survival rate (SR) were calculated using the following formula: WGR (%) = 100 × (W_t_-W_0_) / W_0_; SGR (%) = 100 × (lnW_t_ − lnW_0_) / 30; SR (%) = 100 × (final number of test fish) / (initial number of test fish).

### Non-specific immune analysis

After 30 days of feeding, 3 crucian carps were randomly selected from each group for tail vein blood sampling. The blood was coagulated at 4 °C for 12 h, and the upper serum samples were collected. The activities of acid phosphatase (ACP), alkaline phosphatase (AKP), peroxidase (CAT), and superoxide dismutase (T-SOD) were determined by kit (Nanjing Jiancheng Institute).

### Attack test

After 30 days of feeding, 30 fish in each group were intraperitoneally injected with 0.2 mL *A. hydrophila* bacterial suspension. In addition, the crucian carp in the blank control group was intraperitoneally injected with PBS 0.2 mL, and the mortality of crucian carp was observed and recorded every day for 7 days.

### Colonization of strain L-82^EGFP^ in crucian carps and its interaction with A.HX040

The ET12567/PUZ8002EGFP plasmid in *E. coli* was transferred into strain L-82 by conjugation transfer technology to construct an L-82^EGFP^ fluorescently labeled strain. The recombinant strain was added to the feed at a concentration of 1 × 10^8^ CFU/mL and fed to crucian carp for 14 days (*n* = 20). The distribution and colonization of the fluorescent strain L-82^EGFP^ in crucian carp were observed with a small animal in vivo imaging system (IVIS, Caliper, USA) every other day.

Crucian carps with similar size and health were selected and divided into two groups on average, with 10 fish in each group. The replicates were set up. Crucian carps were starved and domesticated without feed 48 h before the experiment. At the beginning of the experiment, AhX040^mCherry^ was injected into the crucian carp by intraperitoneal injection, with a dose of 200 μL per tail. The small animal in vivo imaging system was used to observe for 7 days. On the 7th day, the strain L-82 with a concentration of 1 × 10^8^ CFU/mL was added to the feed of the experimental group, and the small animal in vivo imaging system was used to observe the distribution of AhX040^mCherry^ in the experimental group compared with the control group every other day.

### Whole genome sequencing of L-82

The strain L-82 was cultured in AM3-1 medium to logarithmic phase, and the bacteria were collected, washed with sterile water and frozen in liquid nitrogen, and then sent to Nextomics Biotechnology Co., Ltd (Wuhan, China) for whole genome sequencing. Genomic DNA of L-82 was extracted using the Qiagen kit and purified using magnetic beads (0.5 ×). After the genomic DNA of L-82 was quantified by NanoDrop and Qubit, the whole genome was sequenced by PromethION. The sequencing data after quality control was assembled by Flye, calibrated by Pilon, and optimized using a circulator. Functional annotations were predicted using the KEGG, GO COG, and RAST databases, and a circular map of the L-82 genome was performed using Circos.

### Separation and purification of antibacterial active substances

The fermentation supernatant was collected, and the antibacterial active substances were extracted with ethyl acetate as the extractant and 100% methanol as the solvent. The antibacterial experiment of *A. hydrophila* was carried out by filter paper method to detect the extraction and purification effect. The crude extract was separated and purified by high-performance liquid chromatography (Agilent 1260) using a YMC-Pack ODS-AQ reversed-phase C18 column. The separation product with the best bacteriostatic effect was selected and detected by liquid chromatography linear ion hydrazine mass spectrometry system (Thermo LTQ Orbitrap XL). The temperature was set at 30 °C, ESI was used as the ion source, the voltage was 4000 V, and helium was used as the collision gas for a full scan. According to the mass-to-charge ratio obtained after mass spectrometry detection and the predicted molecular formula, the antibacterial active substances were identified.

### Statistical analysis

All data were statistically analyzed using SPSS 20 software and expressed as mean ± standard error of mean (SEM). Independent-sample *t*-tests and least significant difference test were used to determine the difference (*P* < 0.05).

## Result

### Isolation and identification of L-82

**Fig. 1:**
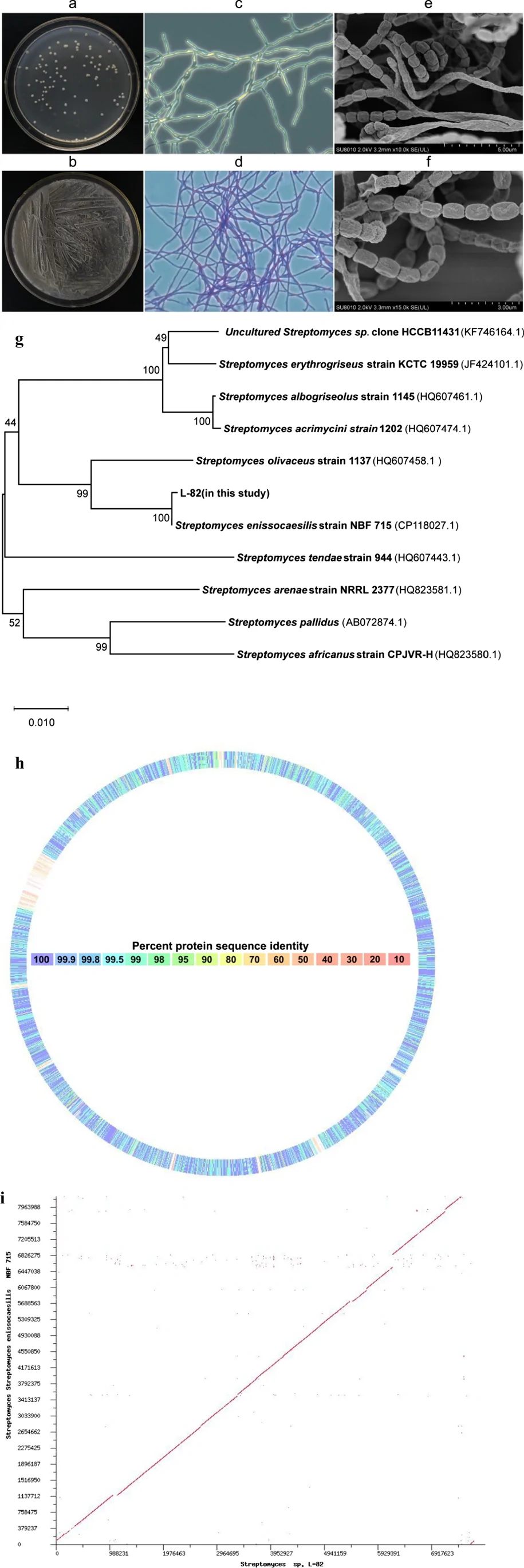
Identification of strain L-82. **a** The plate colony morphology of strain L-82. **b** The plate spores morphology of strain L-82. **c** Morphology of strain L-82 under phase contrast microscope (1000 ×). **d** The gram-straining of strain L-82. **e** Strain L-82 (10,000 ×). **f** Strain L-82 (15,000 ×). **g** Phylogenetic tree of strain L-82 based on *gyrB* gene sequence. **h** Sequence similarity between strain L-82 and *Streptomyces enissocaesilis* strain NBF 715. **i** Genome-wide synteny analysis of strain L-82 and *Streptomyces enissocaesilis* strain NBF 715

An antagonistic actinomycete was isolated from the soil of Xinyang City, Henan Province, which had inhibitory effect on 10 kinds of fish pathogens such as *A. hydrophila* and *A. veronii* (Fig. S1 and Table 1). The colony morphology (Fig. 1a, b, c), Gram staining characteristics (Fig. 1d), and physiological and biochemical characteristics (Tab. S1) of the strain were in line with the typical characteristics of Actinomycetes. Optical microscope and scanning electron microscope showed that the strain L-82 had developed hyphae, more branches, flexible spore filaments, long round spores, and rough surface (Fig. 1e, f).

**Table 1:** The antimicrobial spectrum of strain L-82

Strain	Inhibition zone diameter (mm)
Shewanella xiamenensis	27.50 ± 0.25
Erwinia spp.	26.60 ± 0.29
Aeromonas hydrophila	22.80 ± 0.63
Aeromonas caviae	22.40 ± 0.29
Aeromonas allosaccharophila	20.58 ± 0.29
Citrobacter freundii	21.40 ± 0.38
Edwardsiella tarda	18.10 ± 0.52
Aeromonas sobria	19.75 ± 0.43
Plesiomonas shigelloides	18.10 ± 0.29
Aeromonas veronii	17.40 ± 0.76

The strain L-82 was most closely related to *Streptomyces enissocaesilis* strain NBF 715 (Fig. 1g). The partial sequence similarity of the whole genome of the two strains was low, only about 50% (Fig. 1h), and not completely collinear(Fig. 1i). The strain L-82 was identified as a new strain of *Streptomyces enissocaesilis*.

### Antibacterial ability and stability *in vitro*

**Fig. 2:**
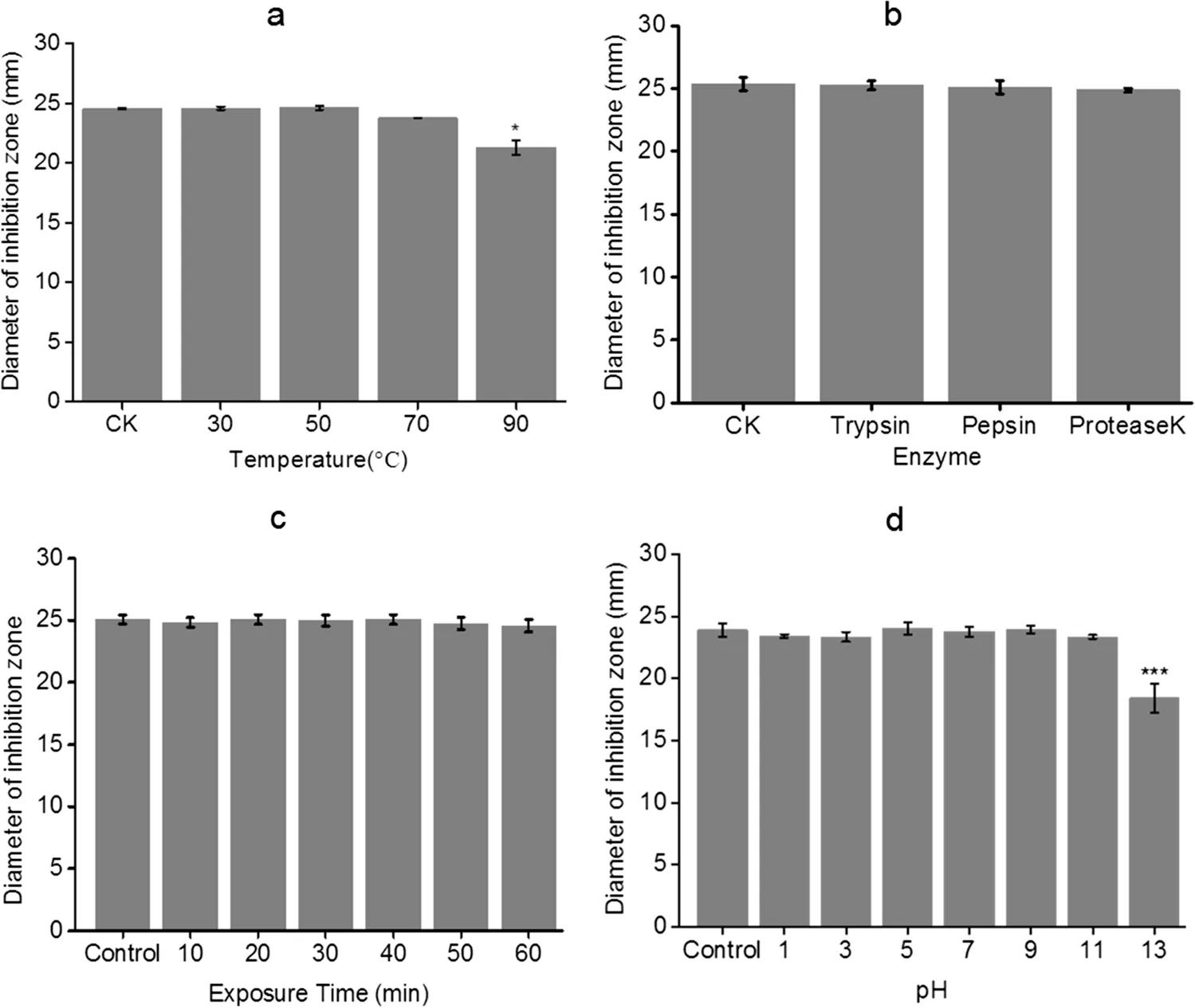
Antibacterial activity of fermentation broth of strain L-82 treated under different conditions against *A. hydrophila*. **a** The diameter of the inhibition zone of the fermentation broth treated at different temperatures against *A. hydrophila*. **b** The diameter of the inhibition zone of the fermentation broth treated with different enzymes against *A. hydrophila*. **c** The diameter of the inhibition zone of the fermentation broth after UV irradiation on *A. hydrophila*. **d** The diameter of the inhibition zone of the fermentation broth treated with different pH on *A. hydrophila*

The antibacterial effect of strain L-82 fermentation broth with different fermentation times on *A. hydrophila* was detected. The results showed that the 6th day was the best fermentation time (Fig. S2). The MIC and MBC values of the fermentation broth on the 6th day were determined, and the results were 64 times and 16 times the concentration of the fermentation broth (Tab. S2). This indicates that the strain L-82 has good antibacterial properties in vitro. In order to detect the antibacterial stability of the fermentation broth, the fermentation broth of strain L-82 was treated with different temperatures, proteases, pH, and ultraviolet irradiation time. The results showed that the antibacterial active substances produced by the strain had a certain tolerance to high temperature, protease, pH, and UV, and showed high stability against *A. hydrophila* (Fig. 2).

### Security evaluation

**Fig. 3:**
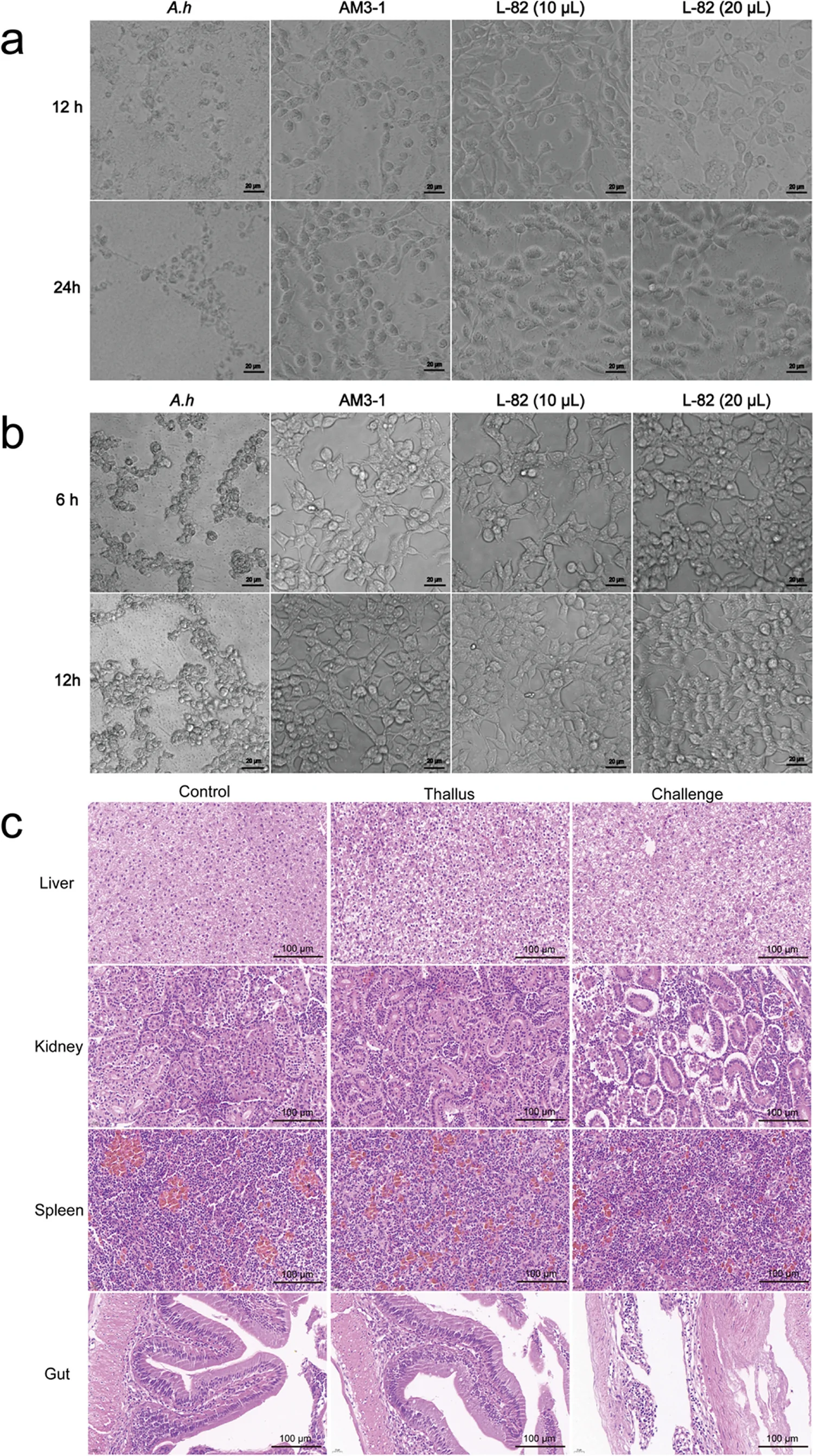
Safety evaluation of strain L-82. **a** Effect of fermentation supernatant of strain L-82 on liver cells of crucian *auratus.*AM3-1: medium (negative control); *A.h*: cell-free culture supernatant of *A. hydrophila* (positive control); L-82 (10 μL): 10 μL of cell-free culture supernatant of strain L-82; L-82 (20 μL): 20 μL of cell-free culture supernatant of strain L-82. **b** Effect of fermentation supernatant of strain L-82 on HEK293T of crucian *auratus*. AM3-1: medium (negative control); A.h: cell-free culture supernatant of *A. hydrophila* (positive control); L-82 (10 μL): 10 μL of cell-free culture supernatant of strain L-82; L-82 (20 μL): 20 μL of cell-free culture supernatant of strain L-82. **c** The effect of strain L-82 on the tissue structure of crucian carp after feeding

In order to use strains for disease prevention and control in fish farming, its biosafety must be evaluated first. The cell morphology of grass carp liver L8824 cells and HEK293T was observed at 12 h and 24 h after treatment with cell-free supernatant of strain L-82. Compared with the cells in the pathogen treatment group, the cells grew well, the morphology was full, and the outline was clear. This indicates that strain L-82 has no in vitro cytotoxicity to L8824 and HEK293T **(**Fig. 3a, b**)**.

In order to evaluate the in vivo safety of the strain, crucian carp was continuously fed with a diet containing strain L-82 (1 × 10^8^ CFU/mL) for 30 days. After feeding, the four immune tissue structures of the liver, kidney, spleen, and intestine of crucian carp were observed. Compared with the normal tissue state of the control group, after the injection of *A. hydrophila*, the renal cell gap of the crucian carp increased, the structure was loose, the small intestinal wall became thinner, the gap between the epithelial cells increased, and the small intestinal villi were relatively normal. The stiff state showed obvious damage. However, the structure of the four immune tissues of crucian carp in the experimental group fed with the feed added with strain L-82 did not change significantly (Fig. 3c). It shows that the strain L-82 will not cause damage to the immune tissue of crucian carp, which proves the safety of the strain in crucian carp.

### Dynamic distribution and protective ability of strain L-82^EGFP^ in crucian carp

**Fig. 4:**
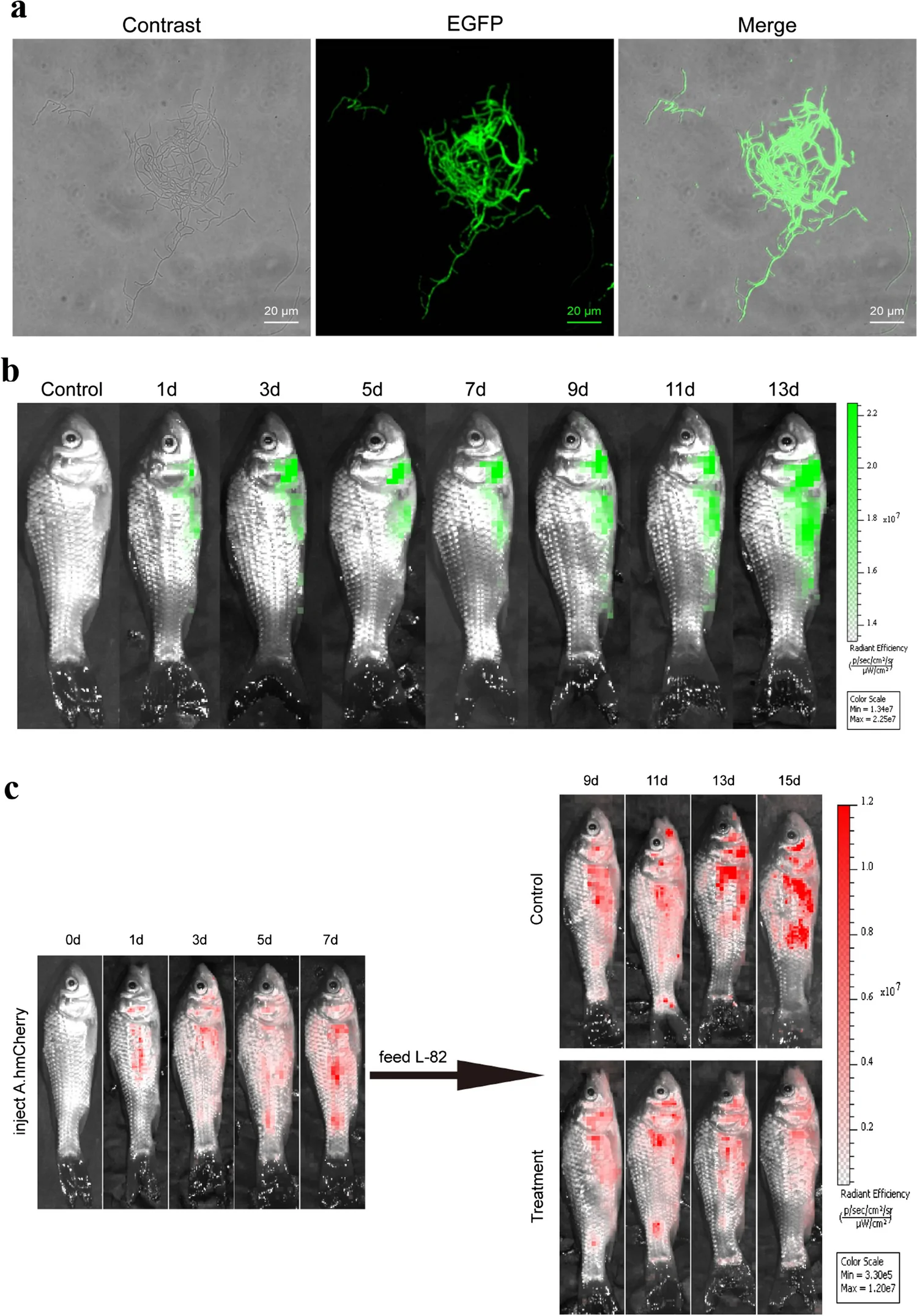
Distribution of strain L-82 in crucian carp and its antagonistic effect against *A. hydrophila*. **a** Morphology of strain L-82^EGFP^ under the fluorescence microscope. **b** The distribution of the L-82^EGFP^ strain in crucian carp was detected using IVIS. **c** The distribution of the AhX040^mCherry^ strain in crucian carp was detected using IVIS

In order to detect whether it can colonize in crucian carp, the L-82^EGFP^ strain was constructed by conjugal transfer technology, and the luminescence was observed by an inverted fluorescence microscope. It can be observed that the labeled strain can emit green fluorescence (Fig. 4a). The growth curves of strains L-82 and L-82^EGFP^ were determined by dry weight method, and the growth trend of the labeled strains was basically consistent with that of the original strains (Fig. S3). Crucian carps were continuously fed with strain L-82^EGFP^ for 14 days and observed with a small animal imaging system every other day. It can be observed that the green fluorescence signal in crucian carp gradually increased with time, indicating that the strain L-82 can survive and colonize stably in crucian carp (Fig. 4b).

In order to detect the antibacterial ability of strain L-82 in crucian carp, the strain AHXO40 ^mCherry^ was injected intraperitoneally into crucian carp, and the strain L-82 was fed after 7 days. Compared with the control group, the red fluorescence signal in the treatment group was weakened, indicating that the strain could inhibit the proliferation of pathogenic bacteria in crucian carp and resist the infection of pathogenic bacteria in crucian carp (Fig. 4c).

### Effect of strain L-82 on growth performance and non-specific immunity of crucian carp

**Fig. 5:**
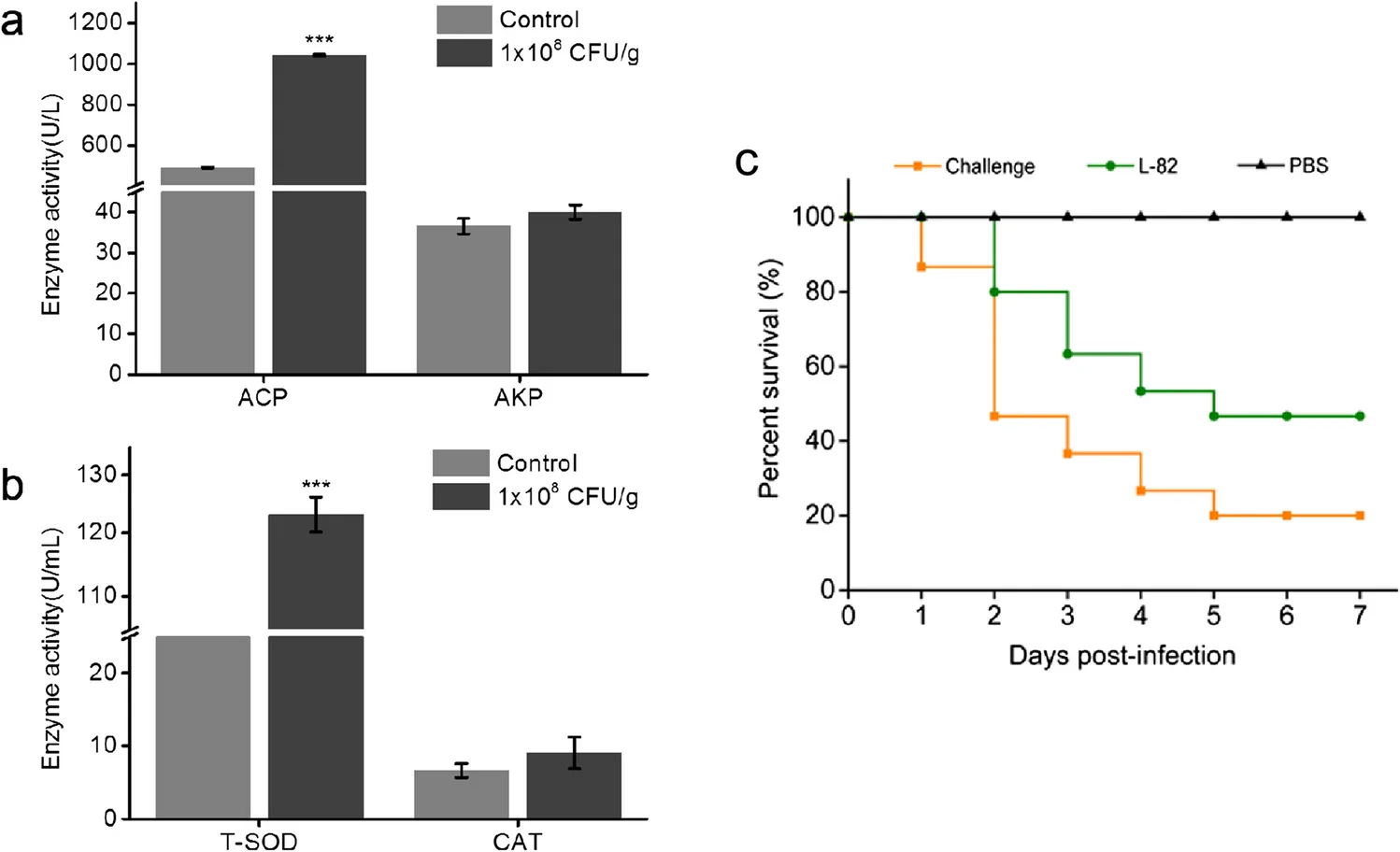
Effects of feeding strain L-82 on non-specific immune indexes in serum of crucian carp (***means *p* < 0.001). **a** Acid phosphatase and alkaline phosphatase activity. **b** Superoxide dismutase enzyme and catalase activity. **c** Cumulative survival of crucian carp challenged with *A. hydrophila*

After feeding, the growth performance of crucian carp was measured. Compared with the control group, the weight growth rate of crucian carp in the feeding group increased by 5.75%, and the specific growth rate also increased (Table 2). The results showed that strain L-82 could enhance the growth performance of crucian carp. The non-specific immune indexes (ACP, AKP, T-SOD, CAT) in the serum of two groups of crucian carp were detected by enzyme activity detection kit. The results showed that compared with the control group, the activities of ACP and SOD in the serum of the feeding group were enhanced (Fig. 5a, b). The results showed that strain L-82 could enhance the immune and antioxidant properties of crucian carp.

**Table 2:** Growth performance of crucian carp

Peer group	*W*_0_ (g)	*W*_t_ (g)	WGR (%)	SGR (%)	SR (%)
Basal feed	10.22 ± 0.08	12.28 ± 0.09	20.20 ± 0.26	0.61 ± 0.007	100
1 × 10^8^ CFU/g	10.26 ± 0.08	12.91 ± 0.06***	25.95 ± 0.71**	0.77 ± 0.019*	100

^*^Means *p* < 0.05,**means *p* < 0.01,***means *p* < 0.001

### Protective ability of strain L-82 to crucian carp

Finally, two groups of crucian carp were challenged with *A. hydrophila*, and the survival rate of crucian carp within 7 days was recorded. The results showed that the survival rate of the strain-feeding group was 26.67% higher than that of the control group. It can be seen that the strain L-82 can enhance the resistance of crucian carp to *A. hydrophila* and protect the survival of crucian carp (Fig. 5c).

### Genome structure prediction and functional annotation of strain L-82

**Fig. 6:**
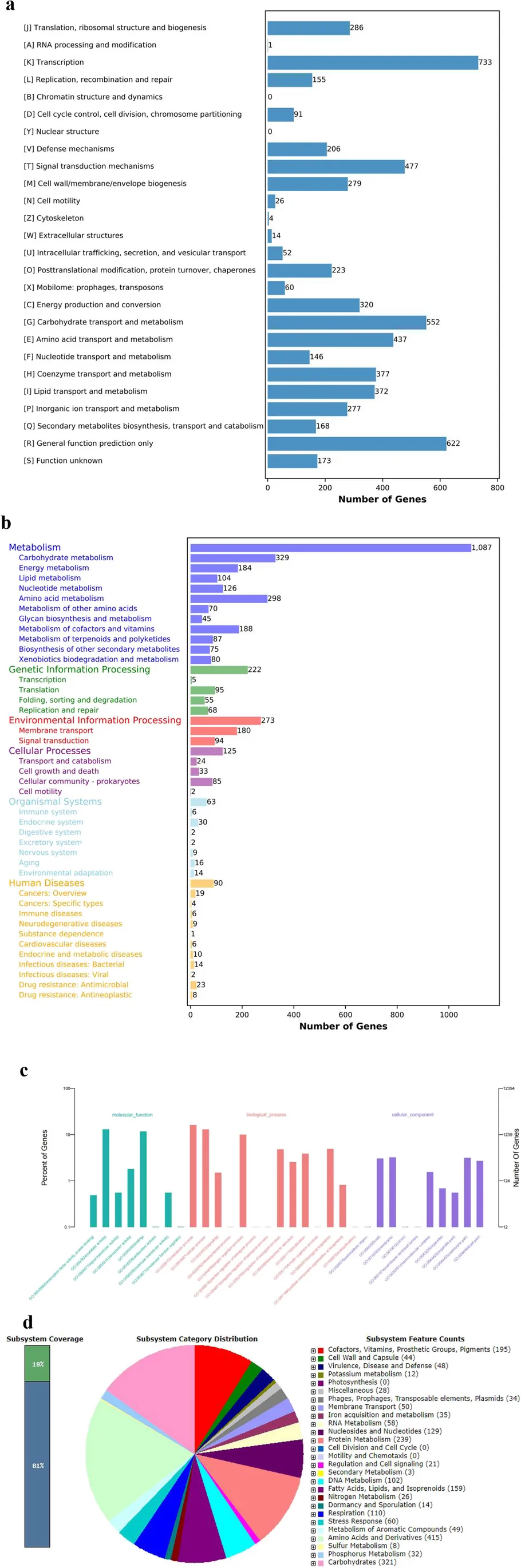
Functional annotation of protein encoded by strain L-82. **a** COG functional classification. **b** KEGG functional classification. **c** GO functional classification. **d** RAST functional classification

The whole genome of strain L-82 was sequenced. The genome size of strain L-82 was 8,592,885 bp, and the GC content was 71.57%. A total of 7402 CDS, 119 tRNA, 6 sets of 16S/23S/5S ribosomal RNA, and 1 genomic island (Tab. S3) were predicted. The genes of strain L-82 were functionally annotated by GO, COG, KEGG, and RAST databases. The results showed that the transcription, metabolism, catalysis, and membrane transport activities of the strain were more active (Fig. 6).

### Prediction of secondary metabolite synthesis gene cluster

The secondary metabolite gene clusters in strain L-82 were predicted by antiSMASH platform, and 27 secondary metabolite gene clusters were obtained. Among them, the matching degree with isorenieratene, 5-dimethylallylindole-3-acetonitrile, streptothricin, flaviolin, ectoine, geosmin, hopene, and SapB reached 100% (Table 3). Isorenieratene is an antioxidant with natural activity and can inhibit light/ultraviolet damage (Chen et al. 2019). 5-Dimethylallylindole-3-acetonitrile is an indole compound. Indole derivatives have been shown to have antibacterial, antiinflammatory, antiviral, antioxidant, antitumor, and other biological activities. Streptothricin has broad-spectrum antibacterial and antifungal activity (Burckhardt and Escalante-Semerena 2017). Flaviolin is a pigment that may protect microorganisms from ultraviolet radiation (Ozaki et al. 2013; Redenbach et al. 1996; Zhao et al. 2005). SapB is a kind of wool thiopeptide. Studies have shown that actinomycete-derived wool thiopeptide has strong antibacterial activity, as well as antitumor, antivirus, and other biological activities. Ectoine is a cyclic amino acid derivative that protects microorganisms from harsh environments such as high salt, high osmotic pressure, drying, and ultraviolet radiation (Ma et al. 2022). The results showed that the synthetic gene clusters of various antibacterial and fungal activities and tolerance to extreme environmental substances were predicted in the genome of strain L-82, and it was speculated that the strain had the potential to synthesize such substances.

**Table 3:** Statistics of gene clusters of secondary metabolites in the genome of strain L-82

Region	Type	Most similar known cluster	Similarity (%)
Region 1.1	Indole, terpene	Isorenieratene	100
Region 1.2	T1PKS, NRPS, NRPS-like, hglE-KS	Candicidin	95
Region 1.3	Terpene	Lysolipin I	4
Region 1.4	Indole	5-dimethylallylindole-3-acetonitrile	100
Region 1.5	Terpene	Carotenoid	54
Region 1.6	NRPS-like	Streptothricin	100
Region 1.7	T3PKS	Flaviolin/1, 3, 6, 8-tetrahydroxynaphthalene	100
Region 1.8	Ectoine	Ectoectoine	100
Region 1.9	Melanin	Istamycin	4
Region 1.10	Siderophore	Desferrioxamin B/desferrioxamine E	83
Region 1.11	NRPS-like	Alanylclavam/2-hydroxymethylclavam/2-formyloxymethylclavam/clavam-2-carboxylate	12
Region 1.12	Furan, lanthipeptide-class-v	Methylenomycin A	9
Region 1.13	Lanthipeptide-class-iii	Catenulipeptin	60
Region 1.14	Terpene	Julichrome Q3-3/julichrome Q3-5	32
Region 1.15	T2PKS	Spore pigment	66
Region 1.16	NI-siderophore	-	
Region 1.17	Terpene	Geosmin	100
Region 1.18	NI-siderophore	Paulomycin	11
Region 1.19	NRPS	Lipopeptide 8D1-1/lipopeptide 8D1-2	86
Region 1.20	Terpene	Hopene	100
Region 1.21	T1PKS	Vicenistatin	90
Region 1.22	Terpene	Versipelostatin	5
Region 1.23	RiPP-like	Informatipeptin	57
Region 1.24	NRPS	Borrelidin	41
Region 1.25	Lanthipeptide-class-i	Tetronasin	3
Region 1.26	Lanthipeptide-class-iii	SapB	100
Region 2.1	T2PKS, butyrolactone	Fluostatins M-Q	67

### Isolation and identification of antibacterial active substances

**Fig. 7:**
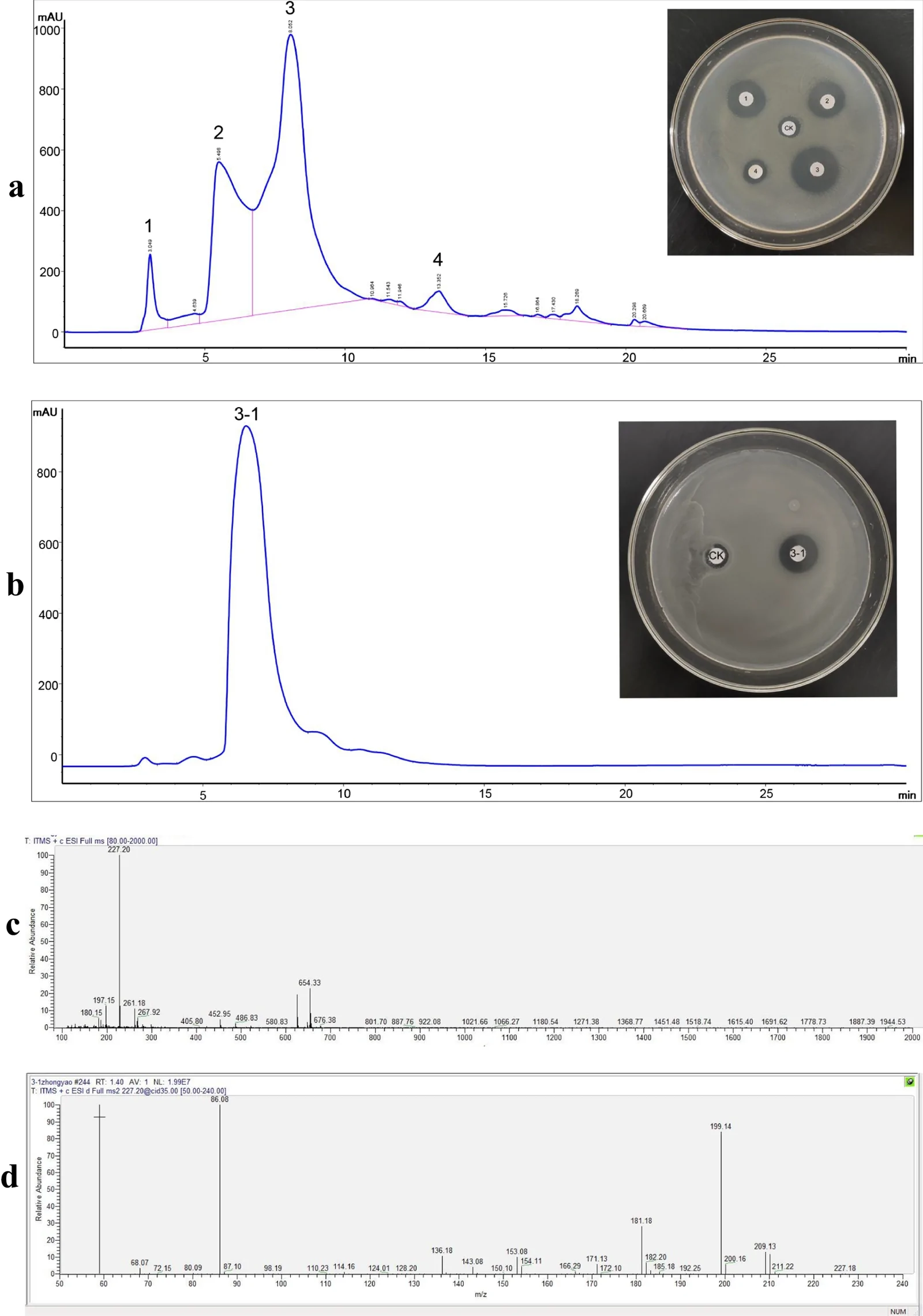
Separation and purification of antibacterial active substances. **a** Isolation of antibacterial active substances from strain L-82. **b** Purification of peak 3. **c** First-order mass spectrometry of peak 3–1. **d** Secondary mass spectrometry of peak 3–1

In order to identify the type of antibacterial active substances of strain L-82, the antibacterial active substances of strain L-82 were separated and purified twice by high-performance liquid chromatography (HPLC), and the active peak 3–1 was collected for mass spectrometry identification. A substance with a molecular mass-to-charge ratio of 227.20 [M + H] ^+^ was detected, and its molecular formulas were predicted to be C_10_H_23_N_6_, C_12_H_25_ON_3_, and C_14_H_27_O_2_. The molecular formula was searched using the PubChem database, and combined with the antiSMASH prediction results, it was speculated that the molecule was a derivative of 5-dimethylallylindole-3-acetonitrile (Fig. 7).

## Discussion

The disadvantages of chemical drug methods for controlling fish diseases have been highlighted, and the development of eco-friendly methods is crucial. Antagonistic bacteria with probiotic functions can fight against diseases, promote growth, and stimulate the host’s immune response to infection. Because of their complex functions, these bacteria are currently favored by researchers. In this study, an actinomycete strain with a broad-spectrum antibacterial effect on fish pathogenic bacteria was screened and isolated from soil samples in Xinyang, Henan Province. The strain was preliminarily identified as a new strain of *Streptomyces enissocaesilis* and named *Streptomyces enissocaesilis* L-82. At present, there are few studies on *Streptomyces enissocaesilis*. Khalel et al. (2020) isolated a strain of *Streptomyces enissocaesilis* AM1 that can degrade keratin in feathers. Manikkasundaram et al. (2022) prepared a biosurfactant from *Streptomyce*s *enissocaesilis* HRB1 to characterize it and evaluate its biomedical and bioremediation potential. Chen et al. (2016) found that the culture filtrate of *Streptomyces enissocaesilis* could significantly reduce the seed germination rate of sunflower rhizosphere weeds (O. cumana). Singh et al. (2020) isolated a strain of *Streptomyces enissocaesilis* capable of producing glucose isomerase from the soil of Madhya Pradesh, India. However, the study of *Streptomyce*s *enissocaesilis* as a probiotic antagonistic bacteria and as a feed additive for fish disease control has not been reported.

The minimum inhibitory concentration (MIC) and minimum bactericidal concentration (MBC) of the fermentation broth of *Streptomyce*s *enissocaesilis* L-82 against *A. hydrophila* were 64 times and 16 times diluted, respectively. This indicated that strain L-82 had a strong antibacterial ability against *A. hydrophila* and had the potential to be developed into an antibacterial agent for fish disease control.

To use strains in fish farming, its biosafety must be evaluated. In this study, the safety of strain L-82 was evaluated from both in vitro and in vivo experiments. Strain L-82 met the safety indicators of feed additives. The strain L-82 screened in this study can tolerate high temperatures below 90 °C and maintain good antibacterial activity under the conditions of strong acid and alkali and ultraviolet irradiation, and its antibacterial effect will not be affected by proteases. Therefore, the strain can be used for the prevention and control of fish diseases in the breeding process.

Some strains can inhibit the colonization of pathogens through competitive exclusion, which may be due to competition of binding sites, synthesis of antibacterial compounds, immune stimulation, or competition of nutrients (Wuertz et al. 2021). Therefore, the colonization ability is a key index to evaluate the antibacterial ability. In this study, it was proved that strain L-82 could stably colonize crucian carp and inhibit the growth of *A. hydrophila* by fluorescent protein labeling technology, which had a protective effect on crucian carp.

In this study, the effects of strain L-82 on the growth, antioxidant, and immune performance of crucian carp were evaluated by analyzing the weight change and immune-related factor levels of crucian carp after feeding strain L-82. It was found that strain L-82 could enhance the weight growth rate of crucian carp. The activities of ACP and SOD in the serum of crucian carp in the experimental group were significantly enhanced. At the same time, the challenge experiment showed that strain L-82 could improve the survival rate of crucian carp after infection with *A. hydrophila*. The results showed that strain L-82 could enhance the growth, antioxidant, and immune performance of crucian carp. This result provides a theoretical basis for the enhancement of the disease resistance of the strain. The synthetic gene clusters of various antibacterial, fungal activities and tolerance to extreme environmental substances were predicted in the genome of strain L-82. It was speculated that the strain had the potential to synthesize such substances, which provided strong evidence for the strain’s antifish pathogen activity at the genetic level, and also laid the foundation for the subsequent exploration of the mechanism of the strain.

Through the separation and mass spectrometry analysis of the antibacterial active substances of strain L-82, a substance with a mass-to-charge ratio of 227.20 [M + H]^+^ was identified. Combined with the prediction results of the secondary metabolite gene cluster, it was speculated that the substance may be a derivative of 5-dimethylallylindole-3-acetonitrile. 5-dimethylallyl indole-3-acetonitrile belongs to indole compounds. Indole derivatives have been proven to have antibacterial, antiinflammatory, antiviral, antioxidant, antitumor, and other biological activities. The result provides a basis for the antibacterial and antioxidant effects of strain L-82 and also provides a direction for the subsequent identification of the structure of the active substance.

In summary, a new strain of *Streptomyces enissocaesilis* L-82 with a broad-spectrum antibacterial effect on fish pathogenic bacteria was screened in this study, which enriched the strain resources. The fermentation broth of strain L-82 has good antibacterial stability and has the potential to tolerate various conditions in practical application. The strain can stably colonize crucian carp and can improve the growth performance, antioxidant activity, immune performance, and disease resistance of crucian carp. Through genome-wide analysis, the metabolic ability and disease resistance potential of the strain were revealed, and it was predicted that the strain had a strong ability to produce secondary metabolites and could be used as a good source of secondary metabolite production. The results of this study provide an effective theoretical basis for the development and utilization of strain L-82. Strain L-82 is expected to be developed into a microbial feed additive to improve the economic benefits of fish farming.

## Supplementary Information

Below is the link to the electronic supplementary material.

Supplementary file1 (PDF 665 KB)

## Data Availability

This manuscript contains previously unpublished data. The name of the repository is not available. The sequencing data have been deposited in the NCBI Sequence Read Archive (SRA) database under the accession code: PRJNA1017178.
