# Supplementary material for: Streptomyces enissocaesilis L-82 has broad-spectrum antibacterial activity and promotes growth for Carassius auratus
Source: Appl Microbiol Biotechnol. 2024 Feb 19;108(1):220. doi: 10.1007/s00253-024-13031-7 (PMC10876771; doi:10.1007/s00253-024-13031-7)
Supplement: Supplementary file 1 — Supplementary file1 (PDF 665 KB) [file 253_2024_13031_MOESM1_ESM.pdf]

Streptomyces enissocaesilis L-82 has broad-spectrum antibacterial activity and promotes growth for *Carassius auratus*

Wensu Long[#1], Wenjuan Zhao[#1], Liangliang He[1], Tahir Ali Khan[1], Ximiao Lai[1],Yunjun Sun[1],

WeitaoHuang[1], Ganfeng Yi[1,2,3*], Liqiu Xia[1*]

1 State Key Laboratory of Developmental Biology of Freshwater Fish, Hunan Provincial Key Laboratory of Microbial Molecular Biology, College of Life Science, Hunan Normal University, Changsha 410081, P. R. China

2 Key Laboratory of Aquatic Functional Feed and Environmental Regulation of Fujian Province, Fujian Dabeinong Aquatic Sci. & Tech. Co., Ltd, Zhangzhou 363500, China

3 Fantastic victory (Shenzhen) scientific innovation group, Co. Ltd.

#These authors contributed equally to this work.

Address for correspondence: College of Life Science, Hunan Normal University, No.36 Lushan Street, Changsha 410081, China, Tel/fax: 86-0731-88872905

E-mail: 2683763570@qq.com, xialq@hunnu.edu.cn.

Tab.S1 The physiological & biochemical reactions of strain L-82

Measuring items	Biochemical reaction	Measuring items	Biochemical reaction
Raffinose	++	Mannitol	+
Galactose	++	Sucrose	+
Inositol	++	Gelatin Liquefaction	-
Fructose	+	H_2S	+
Glucose	+	Hydrolysis of cellulose	-
Xylose	+	Amylohydrolysis	+
Arabinose	+	Glycerol	+
Rhamnose	+		

Tab. S2 Detection of MIC and MBC values of fermentation supernatant of strain L-82 against *A. hydrophila*

Index	Dilution multiple of fermentation broth											
	CK1	CK2	0	2	4	8	16	32	64	128	256	512
MIC	+	-	-	-	-	-	-	-	-	+	+	+
MBC			-	-	-	-	-	+	+	+	+	+

Note: "+" indicates positive; "-" indicates negative

CK1 : LB medium without *A.hydrophila* as negative control ; CK2 : LB medium with *A.hydrophila* as positive control ;

Tab. S3 Statistics of the structure prediction of the genome of strain L-82

Type		Number	Length (bp)	% genome
tRNA		119	9,177	0.11
	16S rRNA	6	9,138	0.11
rRNA	23S rRNA	6	18,727	0.22
	5S rRNA	6	702	0.01
CDS		7,402	7,482,903	87.12
CRISPR		0	0	0.00
Genome_island		1	215,386	2.51

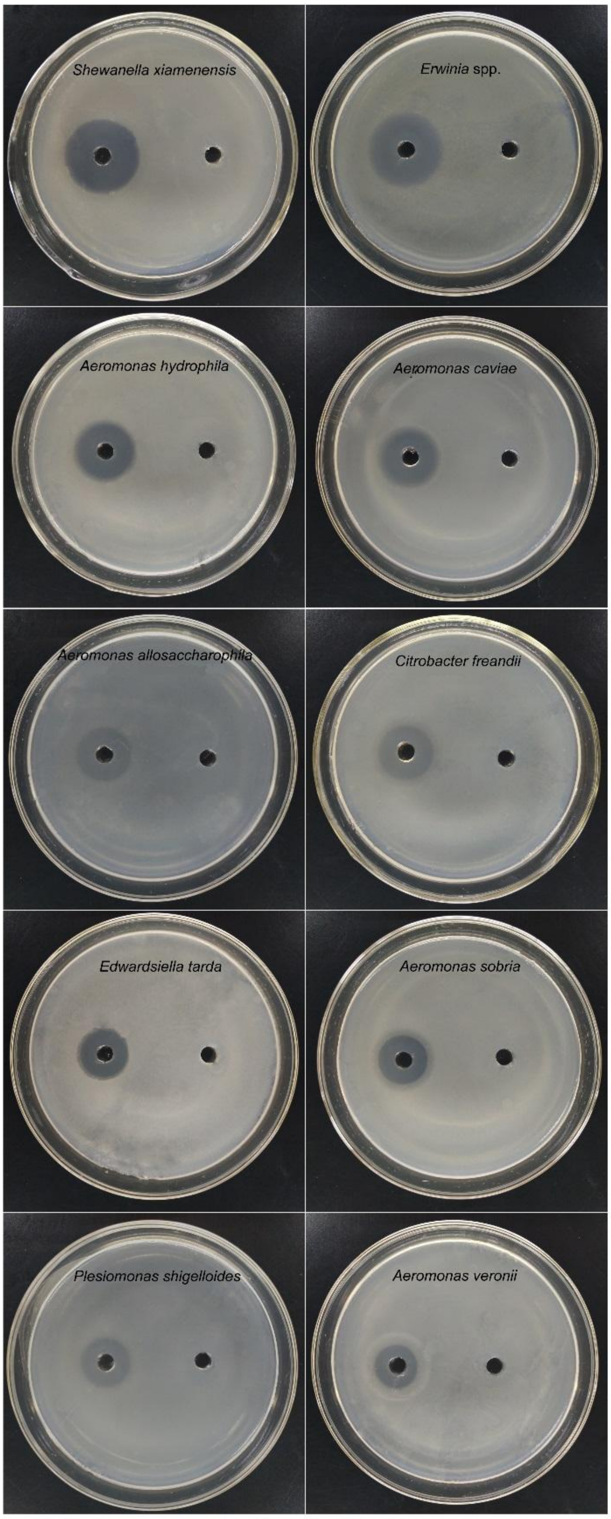

Fig.S1 The antibacterial activity of fermentation supernatant of strain L-82 on fish pathogens

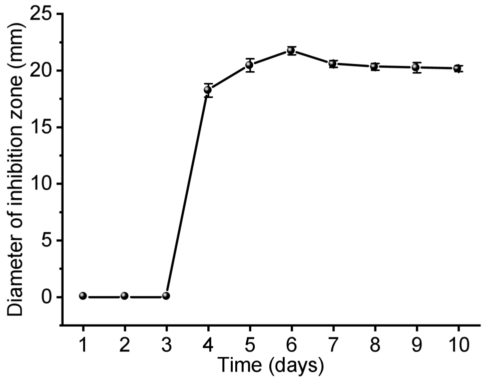

Fig. S2 The antibacterial activity of fermentation supernatant of strain L-82 at different fermentation time on *A. hydrophila*

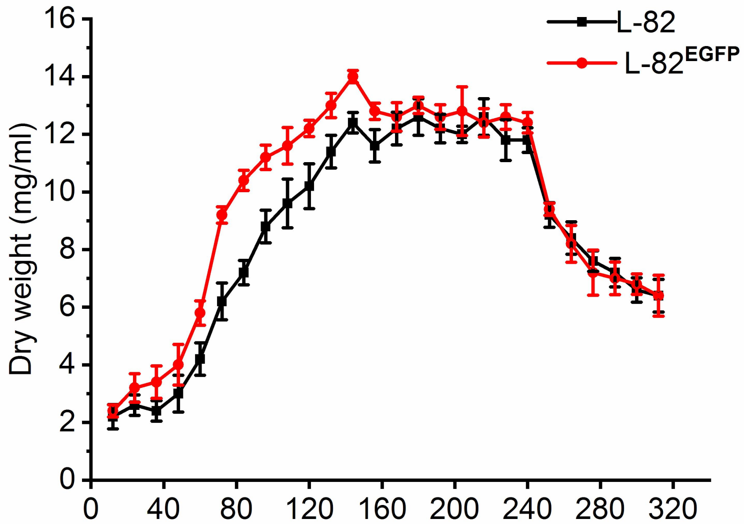

Fig. S3 The growth curve of strain L-82 and strain L-82^{EGFP}
